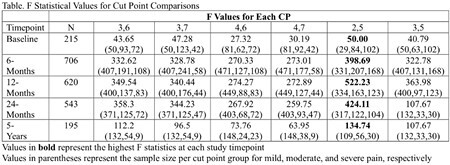# 124 Numerical Cut Points for Mild, Moderate and Severe Pain in Adult Burn Survivors

**DOI:** 10.1093/jbcr/irae036.123

**Published:** 2024-04-17

**Authors:** Gretchen J Carrougher, Alyssa M Bamer, Caitlin M Orton, Maiya I Pacleb, Mary D Slavin, Jeffrey C Schneider, Haig A Yenikomshian, Barclay T Stewart

**Affiliations:** UW Medicine Regional Burn Center, Seattle, WA; University of Washington, Golden, Colorado; UW Medicine Regional Burn Center, Harborview Medical Center, Seattle, WA; Department of Health Law, Policy and Management, Boston University School of Public Health, Boston, MA; Spaulding Rehabilitation Hospital/Harvard Medical School, Boston, MA; University of Southern California, Los Angeles, CA; University of Washington, Seattle, WA; UW Medicine Regional Burn Center, Seattle, WA; University of Washington, Golden, Colorado; UW Medicine Regional Burn Center, Harborview Medical Center, Seattle, WA; Department of Health Law, Policy and Management, Boston University School of Public Health, Boston, MA; Spaulding Rehabilitation Hospital/Harvard Medical School, Boston, MA; University of Southern California, Los Angeles, CA; University of Washington, Seattle, WA; UW Medicine Regional Burn Center, Seattle, WA; University of Washington, Golden, Colorado; UW Medicine Regional Burn Center, Harborview Medical Center, Seattle, WA; Department of Health Law, Policy and Management, Boston University School of Public Health, Boston, MA; Spaulding Rehabilitation Hospital/Harvard Medical School, Boston, MA; University of Southern California, Los Angeles, CA; University of Washington, Seattle, WA; UW Medicine Regional Burn Center, Seattle, WA; University of Washington, Golden, Colorado; UW Medicine Regional Burn Center, Harborview Medical Center, Seattle, WA; Department of Health Law, Policy and Management, Boston University School of Public Health, Boston, MA; Spaulding Rehabilitation Hospital/Harvard Medical School, Boston, MA; University of Southern California, Los Angeles, CA; University of Washington, Seattle, WA; UW Medicine Regional Burn Center, Seattle, WA; University of Washington, Golden, Colorado; UW Medicine Regional Burn Center, Harborview Medical Center, Seattle, WA; Department of Health Law, Policy and Management, Boston University School of Public Health, Boston, MA; Spaulding Rehabilitation Hospital/Harvard Medical School, Boston, MA; University of Southern California, Los Angeles, CA; University of Washington, Seattle, WA; UW Medicine Regional Burn Center, Seattle, WA; University of Washington, Golden, Colorado; UW Medicine Regional Burn Center, Harborview Medical Center, Seattle, WA; Department of Health Law, Policy and Management, Boston University School of Public Health, Boston, MA; Spaulding Rehabilitation Hospital/Harvard Medical School, Boston, MA; University of Southern California, Los Angeles, CA; University of Washington, Seattle, WA; UW Medicine Regional Burn Center, Seattle, WA; University of Washington, Golden, Colorado; UW Medicine Regional Burn Center, Harborview Medical Center, Seattle, WA; Department of Health Law, Policy and Management, Boston University School of Public Health, Boston, MA; Spaulding Rehabilitation Hospital/Harvard Medical School, Boston, MA; University of Southern California, Los Angeles, CA; University of Washington, Seattle, WA; UW Medicine Regional Burn Center, Seattle, WA; University of Washington, Golden, Colorado; UW Medicine Regional Burn Center, Harborview Medical Center, Seattle, WA; Department of Health Law, Policy and Management, Boston University School of Public Health, Boston, MA; Spaulding Rehabilitation Hospital/Harvard Medical School, Boston, MA; University of Southern California, Los Angeles, CA; University of Washington, Seattle, WA

## Abstract

**Introduction:**

The optimal numerical cut points for mild, moderate, and severe burn pain have been established. Investigators at a single center determined that average burn pain can be categorized as mild (0-2), moderate (3-5), and severe (6-10). To address concerns about small sample size and limited diversity, we aimed to identify the most suitable average pain intensity rating scores for mild, moderate, and severe pain in a large, diverse cohort of adult burn survivors using a Patient Reported Outcome Measures Pain Interference (PROMIS-PI) short form.

**Methods:**

Average 11-point pain intensity Visual Analog Scale (VAS) or Numerical Rating Scale (NRS) scores (0-10) and a customized PROMIS-PI 4-question short form were administered to adults with burn injury treated at 5 centers in the US at hospital discharge (baseline) and 6, 12, 24-months and 5-years post-injury. To identify pain intensity scores that best represent mild, moderate, and severe pain, F value statistics associated with multiple ANOVA comparisons for mean pain interference scores by various pain intensity cut points were computed. Based on previous research, 6 possible cut points (CP) were compared: CP 3,6; 3,7; 4,6; 4,7; 2,5; and 3,5. The optimal cut points were considered those with the highest ANOVA F statistic.

**Results:**

Data from 1,190 participants (68% male, 80% white, 22% Hispanic, mean age 46 years) and 5 study centers with VAS/NRS pain intensity and PROMIS-PI scores at one or more study timepoints were analyzed. The optimal classification for mild, moderate, and severe pain was CP 2,5 at all timepoints (see table).

**Conclusions:**

This study confirms that for a diverse cohort of burn-injured adults from multiple centers within the United States, burn intensity ratings of mild (0-2), moderate (3-5), and severe (6-10) is appropriate for up to 5 years post-injury. These findings support previous research findings and continue to advance our understanding of burn pain and pain intensity reporting while providing clinicians and researchers with an objective measure that can be used for screening, treatment, quality improvement, and research.

**Applicability of Research to Practice:**

Use of this numerical classification for burn pain in the adult burn survivor can be used with greater confidence in diverse populations.